# Reply to Comment on Katsarelias, D., et al. “The Effect of Beta-Adrenergic Blocking Agents in Cutaneous Melanoma—A Nation-Wide Swedish Population-Based Retrospective Register Study.” *Cancers* 2020, *12*, 3228

**DOI:** 10.3390/cancers13010092

**Published:** 2020-12-30

**Authors:** Dimitrios Katsarelias, Hanna Eriksson, Rasmus Mikiver, Isabelle Krakowski, Jonas A. Nilsson, Lars Ny, Roger Olofsson Bagge

**Affiliations:** 1 Department of Surgery, Institute of Clinical Sciences, Sahlgrenska Academy, University of Gothenburg, 405 30 Gothenburg, Sweden; dimitrios.katsarelias@gu.se (D.K.); jonas.nilsson@perkins.com.au (J.A.N.); 2 Department of Surgery, Sahlgrenska University Hospital, 413 45 Gothenburg, Region Västra Götaland, Sweden; 3 Department of Oncology and Pathology, Karolinska Institutet, 171 64 Stockholm, Sweden; hanna.eriksson.4@ki.se (H.E.); isabelle.krakowski@sll.se (I.K.); 4 Theme Cancer/Department of Oncology, Karolinska University Hospital, 171 76 Stockholm, Sweden; 5 Regional Cancer Center South East Sweden and Department of Clinical and Experimental Medicine, Linköping University, 581 83 Linköping, Sweden; Rasmus.Mikiver@regionostergotland.se; 6 Theme Inflammation/Department of Dermatology, Karolinska University Hospital, 171 76 Stockholm, Sweden; 7 Harry Perkins Institute of Medical Research, 6009 Nedlands, Australia; 8 Department of Oncology, Institute of Clinical Science, Sahlgrenska Academy, University of Gothenburg, Sahlgrenska University Hospital, 405 30 Gothenburg, Sweden; lars.ny@oncology.gu.se; 9 Wallenberg Centre for Molecular and Translational Medicine, University of Gothenburg, 405 30 Gothenburg, Sweden

We thank De Giorgi et al for their interest in our study, and for raising important and relevant questions. As a general comment, the idea of a positive effect of beta-blockers in reducing recurrence of melanoma is very intriguing [1,2], and therefore our aim was to investigate this in a large cohort of patients. The study was a retrospective registry-based study including 12,738 patients with melanoma, where 3702 were exposed to beta-blockers [3].

Concerning the question of patients with thin melanomas, it is true that including a low-risk group would potentially dilute the observed effect. However, when we excluded low-risk patients (T1 with Breslow thickness less than 1 mm) from the analysis, this did not affect the results. For example, when specifically analyzing melanoma-specific survival (MSS) for patients exposed to selective beta-blockers, divided in three cohorts, i.e., all with melanomas thicker than 1 mm (T2–T4), thicker than 2 mm (T3–T4) and thicker than 4 mm (T4), the HRs were 1.02 (*p* = 0.95), 1.04 (*p* = 0.77) and 1.04 (0.66), respectively. When performing the same analysis for non-selective beta-blockers, the HRs were 0.78 (*p* = 0.26), 0.82 (*p* = 0.41) and 0.64 (*p* = 0.16). This analysis shows that the HR is largely unaffected by risk group and that excluding thin melanomas (T1) does not alter the results.

The question concerning the timing of beta-blocker use, and specifically if patients used beta-blockers also before their melanoma diagnosis, is more complex. We included all patients with melanoma in Sweden during the time-period 2009–2013 (excluding patients with multiple melanomas, stage IV at diagnosis or missing information concerning Breslow thickness or ulceration). For these 12,738 patients, all their prescriptions of beta-blockers were retrieved from the Swedish Prescribed Drug Registry. If patients had previously in life been exposed to beta-blockers, hypothetically then affecting incidence rather than survival, is not known since the registry do not go back that many years. The question concerning exposure time of beta-blockers was indeed analyzed by comparing the above and below median daily defined doses (DDD), and we could not see any beneficial effect of longer exposure. However, the timing of beta-blocker intake was not specifically analyzed, since the analysis is based on prescription data which only gives a rudimentary information on timing.

De Giorgi has previously presented a prospective trial with 53 patients, where 19 received the beta-blocker propranolol, reducing the recurrence rates with 80% [2]. An interesting finding in the current analysis is that although there is no clear overall benefit for selective beta-blockers, there is still a beneficial HR for MSS (0.76 for the whole group), in patients receiving non-selective beta-blockers (i.e., propranolol), but it does not reach statistical significance. This is partly explained by the fact that it was only 365 patients with 24 deaths in the group exposed to non-selective beta-blockers, making any more detailed subgroup analysis impossible. However, we still find this to be an interesting finding, and we are currently aiming at expanding this cohort of patients, and then specifically analyzing the use of propranolol. In this analysis, we will then also include retrospective information concerning previous beta-blocker medication, and we will also try to model in more detail the impact of timing as suggested by De Giorgi et al. [1,2].

In summary, this is a retrospective registry-based study with several limitations, which is clearly stated in the manuscript, and the results have to be interpreted with this in mind. The appropriate way to address the question if there is a benefit of beta-blockers in melanoma would be to perform a prospective study, e.g., a randomized controlled trial, and we would happily support such an effort.

## References

[1] Giorgi V., Grazzini M., Gandini S., Benemei S., Lotti T., Marchionni N., Geppetti P. (2011). Treatment with β-blockers and reduced disease progression in patients with thick melanoma. Arch. Intern. Med..

[2] De Giorgi V., Grazzini M., Benemei S., Marchionni N., Botteri E., Pennacchioli E., Geppetti P., Gandini S. (2017). Propranolol for Off-Label Treatment of Patients with Melanoma: Results from a Cohort Study. JAMA Oncol..

[3] Katsarelias D., Eriksson H., Mikiver R., Krakowski I., Nilsson J.A., Ny L., Olofsson Bagge R. (2020). The Effect of Beta-Adrenergic Blocking Agents in Cutaneous Melanoma—A Nation-Wide Swedish Population-Based Retrospective Register Study. Cancers.

